# Cell-Penetrating Peptides and Supercharged Proteins: A Comprehensive Protocol from Isolation to Cellular Uptake

**DOI:** 10.1021/acs.molpharmaceut.5c01560

**Published:** 2026-02-19

**Authors:** Alexander V. Beribisky, Victoria Sarne, Anna Huber, Markus Hengstschläger, Franco Laccone, Hannes Steinkellner

**Affiliations:** † Institute of Medical Genetics, Center for Pathobiochemistry and Genetics, 27271 Medical University of Vienna, Währinger Straβe 10, Vienna 1090, Austria; ‡ Vienna Doctoral School of Pharmaceutical, Nutritional and Sport Sciences (PhaNuSpo), University of Vienna, Josef-Holaubek-Platz 2, Vienna 1090, Austria

**Keywords:** cell-penetrating peptides, supercharged proteins, intracellular protein delivery, protein characterization, live-cell imaging, imaging flow cytometry

## Abstract

Cell-penetrating peptides (CPPs) and supercharged proteins (SPs) enable efficient intracellular delivery of macromolecules, with expanding applications in basic research and in therapeutic development. Despite their potential, reproducible workflows for isolation, biochemical characterization, and quantitative uptake analysis remain limited. Here, we present a comprehensive and replicable protocol for the isolation, characterization, and cellular uptake analysis of CPP-fusion proteins (CPP-FPs) and SPs using methyl-CpG-binding protein 2 (MeCP2) constructs as a proof-of-principle model. This workflow combines native protein purification with dynamic light scattering (DLS)-based buffer optimization. Cellular uptake is then assessed and quantified under live-cell conditions using high-content imaging and imaging flow cytometry, with additional assays to probe endocytic trafficking routes, identify CPP-like motifs in SPs, and validate transducing CPP-FP/SP functionality. The protein isolation and DLS-guided buffer screen yield samples with long-term stability. Live-cell fluorescence microscopy and imaging flow cytometry enable discrimination between membrane-bound and internalized signal, providing higher accuracy compared to plate-based readouts. MeCP2 sequence probing has revealed the presence of a CPP-like motif that is critical to its internalization. Finally, validation assays clearly demonstrated CPP-FP/SP activity. This protocol integrates advances in protein biochemistry, structural analysis, and live-cell imaging into a reproducible pipeline adaptable to a wide range of CPP- and SP-based protein constructs and provides a practical framework for downstream mechanistic and therapeutic interventions.

## Graphical abstract

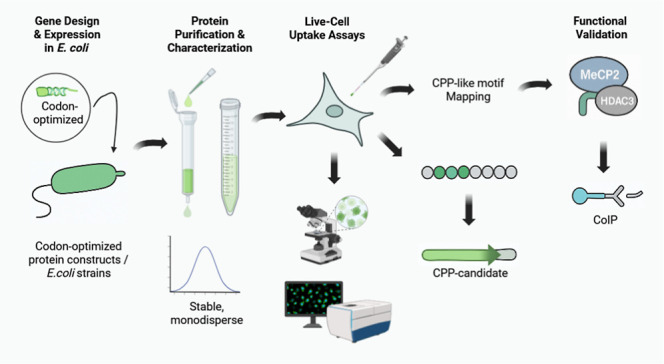

## 1 Introduction

Cell-penetrating peptides (CPPs) are a class of amino acid sequences which are known to possess cell transduction capabilities.^1^ Although the precise mechanisms that underlie CPP-mediated uptake are not fully understood, protein transduction is thought to be triggered by interactions between CPP side chains and the plasma membrane. This is followed by CPP intracellular trafficking, predominantly via endocytosis,^2–4^ though direct CPP translocation has also been reported.^3,5,6^ This membrane-penetrating property has been widely exploited to intracellularly deliver recombinant CPP-fusion proteins (CPP-FPs), both to study intracellular processes^7^ and to support therapeutic development.^8^ One such approach, termed protein replacement therapy, aims to replenish aberrant levels of the intracellular protein of interest brought about by mutational or metabolic disorders. The transactivator of transcription (TAT), an 11-amino-acid CPP derived from HIV-1,^1,9^ has been extensively used for this purpose, enabling efficient intracellular delivery of various therapeutic proteins to treat a number of systemic and neurological disorders.^10–13^

Supercharged proteins (SPs), a class of proteins characterized by an unusually high net charge relative to their molecular weight (CMw), have also emerged as potent tools for cellular delivery. Although many SPs are synthetically engineered, such as GFP variants with net charges ranging from −30 to +36,^14^ several naturally occurring SPs, including β-defensin 3 and c-Jun, have also been reported.^15,16^ SPs participate in a broad range of cellular processes such as gene regulation, signal transduction, and immune response.^17,18^ To mediate these processes, SPs employ not only their various structured domains but also intrinsically disordered regions, which impart unusual resistance to aggregation.^14,17^ Notably, these disordered regions were also shown to mediate SP cellular uptake. Both artificial SPs such as +36 GFP, as well as their naturally derived counterparts, β-defensin 3 and c-Jun, were shown to successfully transduce their mCherry fusions, achieving both cytoplasmic and nuclear delivery.^15,19^

While several methodological studies on CPP-FPs^20^ and SPs^16^ exist, the field is still hampered by the use of unreliable characterization and uptake methodologies^21^ as well as the absence of a streamlined workflow. Recently emerging tools in structural and cell biology, however, now offer a novel, powerful means to improve recombinant protein quality and cellular uptake efficiency. These tools also provide deeper insights into the internalization mechanisms and subcellular compartmentalization of CPP-FPs and SPs. In this work, we present recent methodological advances by comprehensively outlining the sample preparation and uptake experiments (Figure 1), using methyl-CpG-binding protein 2 (MeCP2)-eGFP-derived SP and a CPP-FP variant, termed MeCP2-eGFP (MG) and TAT-MeCP2-eGFP (TMG), as a proof-of-principle model (Figure 2). These recombinant proteins have exhibited clear cellular transduction capabilities with remarkably overlapping sequence and mechanistic features. We anticipate that the methodological insights provided here will support future investigations of CPP-FPs and SP-mediated delivery, with implications for both basic research and therapeutic development.

**Figure 1:**
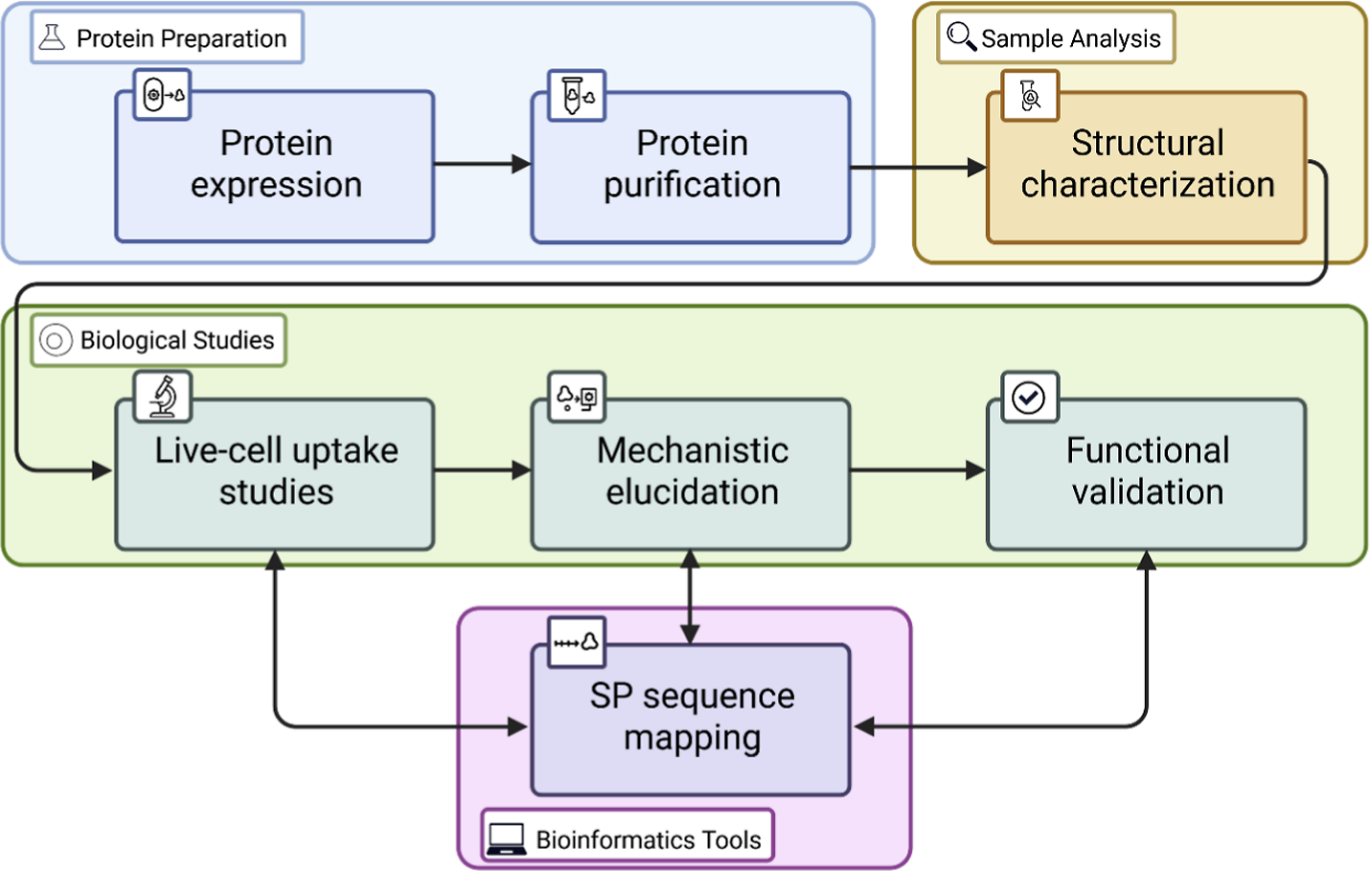
CPP-FP/SP study workflow. A multistep protocol illustrating the expression, purification, characterization, and uptake analysis of CPP-FP/SP, followed by SP-sequence mapping and functional validation.

**Figure 2:**
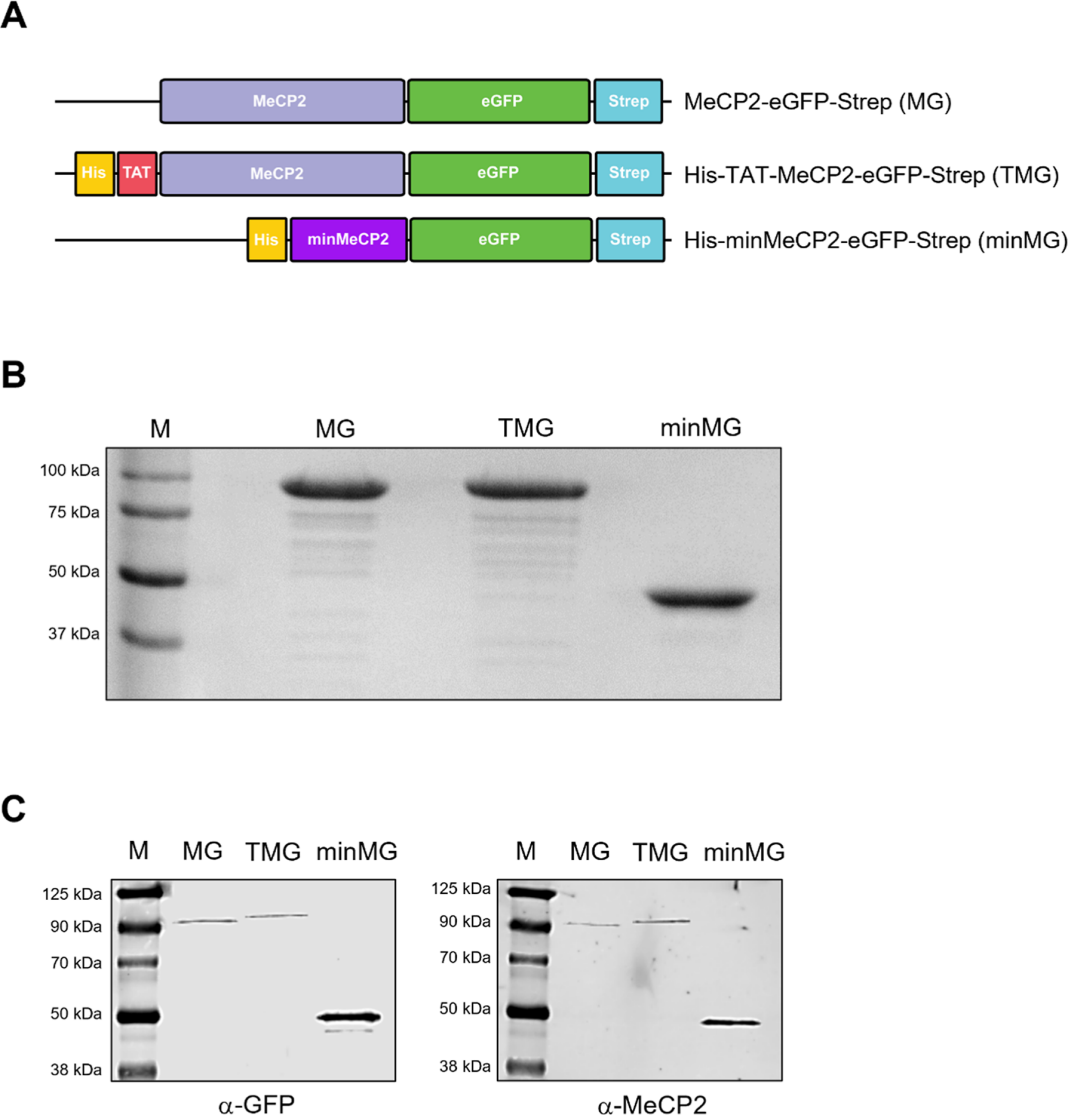
(A) Schematic representation of MeCP2-eGFP, TAT-MeCP2-eGFP, and minMeCP2-eGFP constructs and their appropriate acronyms flanked by a Strep-affinity and His-tags (for TAT-MeCP2-eGFP and minMeCP2-eGFP). (B) A 10% acrylamide SDS–PAGE of MeCP2-eGFP, TAT-MeCP2-eGFP, and minMeCP2-eGFP. M—Precision Plus Protein Dual Xtra protein marker. (C) Western blot of MeCP2-eGFP, TAT-MeCP2-eGFP, and minMeCP2-eGFP stained with the α-GFP (left) and α-MeCP2 (right) antibodies. M—Chameleon DUO prestained marker.

## 2 Materials and Methods

The step-by-step protocol (Supporting Information) presented here consists of three main stages (Figure 1). First, the proteins of interest are expressed in*Escherichia coli* (*E. coli*) and purified using standard chromatographic techniques. The samples are then subjected to a structural analysis with the goal of optimizing buffer conditions conducive to long-term storage. Once an optimal system is selected, uptake studies can commence with live-cell fluorescence microscopy employed for pilot experiments. This is then followed by quantitative imaging techniques such as imaging flow cytometry (IFC) and ImageXpress Pico. These tools can also be used to obtain additional insight into the mechanism of CPP-FP and SP uptake. For the latter, sequence mapping to identify motifs implicated in internalization can also be carried out. Finally, studies to probe internalizing protein activity such as coimmunoprecipitation (CoIP), which are key in confirming CPP-FP/SP functionality, are also demonstrated.

All standard reagents were obtained from commercial sources including Sigma-Aldrich, Thermo Fisher Scientific, and Carl Roth. Additional materials (Table S1) and solutions (Table S2) used in this study are listed in the Supporting Information.

### 2.1 Expression of Recombinant CPP-FPs and SPs

CPP-FPs and SPs are usually expressed in *E. coli* (Table S3) using bacterial expression plasmids such as pET vectors containing the corresponding coding DNA sequence under the control of the *lac* promoter.^22^ The CPP-FP/SP coding DNA fragments should be flanked by affinity tag coding sequences (Figure 2A) to facilitate recombinant protein capture from bacterial cell media.^13,23,24^ To introduce the plasmids into *E. coli*, either chemical transformation or electroporation can be employed. Prior to large-scale protein production, expression conditions should be optimized for each construct to maximize yield and quality. Key parameters include growth temperature, bacterial levels at induction, inducer concentration, expression temperature, and expression time.

### 2.2 Purification of CPP-FPs and SPs

Our CPP-FPs and SPs were purified natively using Strep-TactinXT affinity chromatography, followed by gel filtration chromatography, as a second polishing step (Table S4). Bacterial cells were disrupted by sonication in a Tris-based lysis buffer containing additives to enhance cell lysis and at the same time inhibit proteolysis and maintain protein stability. Following centrifugation to remove cell debris, the clarified lysate was applied to the Strep-TactinXT column. This affinity system was selected due to its gentle elution conditions and ability to yield highly pure protein. As the MeCP2 constructs we purified were nucleic acid binders, they are known to nonspecifically interact with host cell DNA and RNA. To minimize nucleic acid contamination, we treated our CPP-FPs and SPs with a wash buffer containing high amounts of sodium chloride while they were immobilized on the column. This step effectively reduced the *A*_260/280_ ratio of the eluted samples from ∼1.8 to ∼0.6, indicating the successful removal of nucleic acid contaminants. Following affinity purification, gel filtration chromatography (size-exclusion chromatography, SEC) was performed to remove residual degradation products and further enhance purity. Run conditions and buffer composition were optimized in advance using small-scale test samples. During subsequent concentration steps, care must be taken not to reduce the sample volume below defined thresholds, as excessive concentration can promote protein aggregation or precipitation. A critical final step in CPP-FP and SP purification involves lipopolysaccharide (LPS) removal. LPS contamination can induce cytotoxic effects during mammalian cell uptake studies. This is especially relevant for positively charged proteins such as MeCP2, which bind strongly to the negatively charged LPS.^25^ To eliminate LPS, we used a Triton X-114-phase separation method.^26^ For proper extraction, the detergent has to be completely dissolved, with the resulting solution turning turbid. The recombinant protein-Triton X-114 mixture was incubated sequentially on ice at 37 °C and then centrifuged, inducing phase separation into the protein-containing aqueous and the LPS-containing detergent layers. If no such separation occurs, 200 μL of the storage buffer base is added, with the solution vortexed until the detergent is completely dissolved. The aforementioned extraction steps are then repeated. After centrifugation, the aqueous phase was collected, and the extraction was repeated three times for maximum LPS removal. Residual Triton X-114 was subsequently eliminated using a detergent removal column. Prior to use in cellular assays, all protein preparations were evaluated for cytotoxicity using an MTT assay, as recommended.^13,23^

### 2.3 Dynamic Light Scattering of CPP-FPs and SPs

Protein stability was optimized using dynamic light scattering (DLS) experiments carried out on a Wyatt DynaPro II Plate Reader (Wyatt Technology). A buffer screening was conducted to determine the optimal conditions for long-term storage of MG and TMG. Samples were first passed through a 0.1 μm filter membrane and centrifuged at 10,000*g* for 4 min. The resulting supernatant was transferred to a 1.5 mL tube. The protein was diluted to a final concentration of 0.5 mg/mL in each buffer condition listed in Table S5 and dispensed into a 96-well plate. To prevent evaporation, a layer of silicone oil was added on top of each sample. The plate was briefly centrifuged at 500*g* for 1 min to eliminate air bubbles. Measurements were carried out at 25 °C and monitored over the course of 1 week. For measurements under optimal buffer conditions, samples were diluted to a final concentration of 0.5 mg/mL using storage buffer containing 0.05% 3-[(3-cholamidopropyl) dimethylammonio]-1-propanesulfonate (CHAPS). Following the same workflow described above, measurements were conducted at either 37 or 25 °C over a 72 h period. Data analysis was performed by using Dynamics software (Wyatt Technologies).

### 2.4 Live Cell Imaging of CPP-FP and SP-Mediated Protein Transduction

Live-cell imaging of cells transduced with either MG and TMG was carried out according to the protocols outlined in Table S6 and previously published.^13,23,24^ In this study, murine NIH3T3 fibroblasts were used as a model system; however, depending on the protein of interest, a variety of cell lines, including epithelial, fibroblast, and suspension-derived lines (e.g., HeLa, HEK293T), are suitable for these experiments.^11,19^ Recombinant protein concentrations and incubation times should be optimized for each new construct and cell type. As a general starting point, a concentration in a range between 0.5 μM and 5 μM and an incubation period of 1 h are recommended. Endosomal involvement can be investigated by coincubation with endosome-disrupting compounds such as chloroquine (CHQ) or sucrose (Suc), with recommended concentrations of 0.1 and 80 mM, respectively. Conversely, the CPP-FP/SP endocytic trafficking mode can be probed by preincubation with known endocytosis inhibitors such as amiloride (Ami), indomethacin (Ind), and chlorpromazine (0.5 mM each). Following incubation, membrane-adhering (noninternalized) protein must be removed by performing a heparin wash. This step is critical to ensure accurate quantification and visualization of internalized protein during subsequent washing and imaging procedures.^27^

### 2.5 IFC of CPP-FP and SP-Mediated Protein Transduction

The incubation and sample preparation steps for IFC largely follow the live-cell imaging protocol with some key modifications (Table S7). While only NIH3T3 cells were used for CPP-FP/SP studies, IFC demonstrates a broad utility across multiple cell lines.^28–30^ To detach adherent cells from the culture plate, we used 0.05% (v/v) trypsin. It is crucial to avoid prolonged trypsinization, as extended exposure can increase membrane permeability and potentially bias protein uptake.^31^ Following detachment and subsequent processing, cells were imaged immediately to prevent dye diffusion and loss of cell viability, as both skew measurements of protein internalization. Data acquisition was carried out using the ImageStreamX Mark II imaging flow cytometer (Amnis). For cells stained with Hoechst and incubated with eGFP-tagged fusion proteins, the following acquisition channels were used: bright-field imaging, fluorescence excitation with a 405 nm laser for Hoechst detection, and a 488 nm laser for GFP detection.

Image data were analyzed using IDEAS v6.2 software (Amnis). Cells were focused using the gradient root-mean-square bright-field (*Gradient RMS_BF*) function, using local intensity gradients. Focused cells were identified and plotted using area bright-field (*AREA_BF*, based on size) versus aspect ratio bright-field (*Aspect ratio_BF*, based on shape) parameters. The singlets were then gated, and 5000 events were collected per sample. Recombinant protein was evaluated using INSPIRE v201.1.0.693 (Amnis), with the colocalization wizard employed to detect overlapping signals between Hoechst and GFP. Co-localization of Hoechst and GFP signals with subsequent quantification (reported as a median score across cells) was performed using the *Bright Detail Similarity R3_MC_Ch02_Ch07_Median* metric. Experiments were performed in biological triplicates, meaning that three distinct protein samples and three cell batches seeded on different dates were used for three separate measurements. Statistical significance was determined using an ordinary one-way ANOVA, followed by multiple comparison analysis in GraphPad Prism. Further methodological details are available elsewhere.^24^

### 2.6 ImageXpress Pico Uptake Experiments of CPP-FPs and SPs

MG and TMG internalization was further assessed using the ImageXpress Pico imaging system. As with previously described imaging tools, this technique is applicable to multiple cell lines. Sample preparation and processing largely follow the live-cell imaging protocol, with minor adjustments including variations in cell density and a higher Hoechst concentration (Table S8). Parameters such as protein concentration, incubation time, and the use of small molecules (e.g., endosomal disruptors or endocytosis inhibitors) can be carried over from live-cell imaging experiments as an initial reference; however, subsequent empirical optimization should be performed as appropriate. Following image acquisition, nuclear GFP/Hoechst double-positive signals were quantified alongside the total number of nuclei using CellReporterXpress software (Molecular Devices).

### 2.7 Mapping of CPP-like Motifs in SPs

To identify peptide sequences which may be implicated in SP uptake, we employed the CPPSite 2.0 software^32,33^ to scan the primary amino acid sequence of MeCP2 for CPP-like motifs. The highest-scoring candidates identified by the algorithm, which cannot be ruled out based on previous work, were selected for experimental validation (see Table S9). Each candidate motif was cloned as a fusion construct with eGFP, positioned at either the N- or C-terminus. These recombinant CPP-eGFP fusion proteins were then expressed and purified using a protocol largely consistent with that described for full-length CPP-FPs and SPs (Tables S3 and S4), with two notable exceptions: gel filtration chromatography was conducted in DPBS containing 10% glycerol, pH = 7.2 and the LPS removal step was omitted, as these short constructs demonstrated minimal affinity for LPS under the tested conditions. Live-cell imaging experiments were then performed as previously described (Tables S6 and S7). A higher protein concentration of 8–15 μM should be used in these measurements (here, 10 μM) to obtain a sufficient uptake signal. Cell survival at these elevated concentrations has to be verified using a metabolic activity assay (e.g., MTT assay) to exclude uptake due to reduced viability. Variants exhibiting appreciable cellular internalization were flagged as functionally active CPP candidates. To confirm the functional importance of identified CPP motifs, a deletion mutant of the parent SP lacking the candidate sequence (SPΔCPP) was generated and purified under the same conditions (Tables S3 and S4). SPΔCPP uptake was then assessed using live-cell fluorescence microscopy and/or IFC at regular standard SP concentrations (Tables S6 and S7). A marked reduction in SP internalization compared to the wild-type protein would support the role of the deleted motif in mediating cellular uptake.^24^

### 2.8 CoIP of CPP-FP and SP Binding Partners

CoIP studies to assess the binding abilities of internalized MG and/or TMG were carried out as previously described,^24,34,35^ with a number of modifications (Table S10). The protocol involves incubation of murine NIH3T3 fibroblasts with the recombinant proteins, followed by cell lysis and isolation of the cells’ nuclear fraction. In order to capture the MeCP2 complexes via the proteins’ Strep-tag, the samples are then loaded onto Strep-beads, washed, eluted, and loaded on an acrylamide gel. Following electrophoresis and blotting, the membranes are stained with anti-GFP and anti-HDAC3 antibodies to detect MG/TMG and their histone deacetylase 3 (HDAC3) binding partner, respectively. Antibodies for other MeCP2 interactors can also be used. Following secondary antibody incubation, the membranes are imaged.

Several considerations must be taken into account when performing this experiment. Given the high protein amounts required, we recommend a preliminary cytotoxicity screening (e.g., MTT assay) to optimize dosing and avoid unnecessary sample loss. In addition, proper separation between the nuclear and cytoplasmic fractions should be verified by cross-incubation using appropriate markers (e.g., histones and β-tubulin, respectively).^24,34^ Finally, it is essential to include a control in which untreated nuclear isolates are spiked with recombinant CPP-FP/SP.

## 3 Results

### 3.1 Native Purification and Buffer Screening Gives Rise to Soluble, Nonaggregating SPs

Expression and subsequent purification under native conditions yield soluble SPs and CPP-FPs, including MG and TMG. On the SDS-PAGE (Figure 2B) as well as on the Western blot (Figure 2C), these MeCP2 constructs migrate more slowly than expected for a protein of their size, in line with previous observations.^23^ The stability of these proteins can be verified by using a number of tools. Our method of choice is DLS, a native, dye-free technique that enables both the screening of multiple storage conditions and the assessment of long-term stability over several days.^23^ The main parameters to be monitored in such an experiment are sample intensity and hydrodynamic radius (*R*_h_), which serve as proxies for sample stability and aggregation propensity, respectively. An abnormally high *R*_h_ of a given protein sample (i.e., significantly higher than its predicted value) points to the presence of high-molecular-weight aggregates. These abnormal *R*_h_ values can occur upon initial measurements or increase over a certain span, pointing to the time-dependent emergence of protein aggregates. Conversely, a decrease in *R*_h_ and sample intensity, either immediately or over time, strongly indicates protein degradation. As a reporting tool, the ratio of final-to-initial intensity and *R*_h_ is used, with values close to 1.0 indicating minimal change over the measurement period.

To screen for optimal storage conditions, TMG was incubated in multiple buffer solutions in a 96-well plate at 25 °C (Table S5). The selected buffer system consisted of DPBS, 200 mM NaCl, 10% (v/v) glycerol, and 0.05% (w/v) CHAPS, adjusted to pH 7.2. Under these conditions, all constructs remained stable for up to 72 h at 37 and 25 °C, with final-to-initial intensity and *R*_h_ ratios largely being close to 1.0, pointing to high long-term stability and resistance to aggregation (Table 1). This example clearly illustrates the utility of DLS in identifying buffer conditions conducive to long-term CPP-FP and SP stability for their subsequent utilization in downstream experiments.

**Table 1:** MG, TMG, and minMG Intensity Radius Values before and after a 72 h Incubation at 37 and 25 °C in Storage Buffer^a^

construct, 37 °C	parameter	initial value	final value	ratio
MG	intensity	4.4 × 10^6^ Cnt/s	4.6 × 10^6^ Cnt/s	1.05
	radius	6.7 nm	6.4 nm	0.96
TMG	intensity	6.5 × 10^6^ Cnt/s	6.4 × 10^6^ Cnt/s	1.00
	radius	9.1 nm	8.0 nm	0.89
minMG	intensity	4.2 × 10^6^ Cnt/s	3.7 × 10^6^ Cnt/s	0.95
	radius	5.5 nm	4.9 nm	0.90

construct, 25 °C	parameter	initial value	final value	ratio
MG	intensity	3.0 × 10^6^ Cnt/s	2.0 × 10^6^ Cnt/s	0.67
	radius	6.9 nm	6.3 nm	0.91
TMG	intensity	3.3 × 10^6^ Cnt/s	2.81 × 10^6^ Cnt/s	0.85
	radius	8.1 nm	7.8 nm	0.96
minMG	intensity	4.1 × 10^6^ Cnt/s	4.8 × 10^6^ Cnt/s	1.17
	radius	4.4 nm	4.1 nm	0.93

a Adapted from ref 23. Available under CC BY 4.0. Copyright 2022 Springer Nature Link.

### 3.2 Image-Based Live-Cell Analysis Tools Successfully Assess and Quantify CPP-FP/SP Uptake

Once optimal buffer conditions for SPs and CPP-FPs have been established, imaging experiments can be conducted to investigate their potential uptake capabilities. Live-cell imaging has a long-standing history as a key technique for studying protein transduction.^11,15^ Here, this technique was used to visualize MG and TMG (4 μM) internalization into NIH3T3 cells with subsequent accumulation at their heterochromatic foci (Figures 3A and S1), in line with previous findings.^36,37^ A nontransducing version of MeCP2,^24^ termed minMeCP2-eGFP (minMG), isolated (Figure 2) and characterized (Table 1) in the same fashion as MG and TMG, was employed as a negative control, showing no cell-internalizing activity (Figure S1). A crucial intermediate step before imaging is the removal of nonincorporated protein with the glycosaminoglycan heparin. This compound is known to act as a competitive inhibitor of positively charged CPP binding to the cell surface^38^ and was previously successfully employed in multiple live-cell studies.^13,23,24^ In the absence of heparin treatment, significant membrane-adhering protein persists, making the interpretation of cellular uptake highly challenging (Figure 3A). The removal of nontransduced protein significantly improves imaging quality, enabling clearer visualization of MG and TMG uptake.

**Figure 3:**
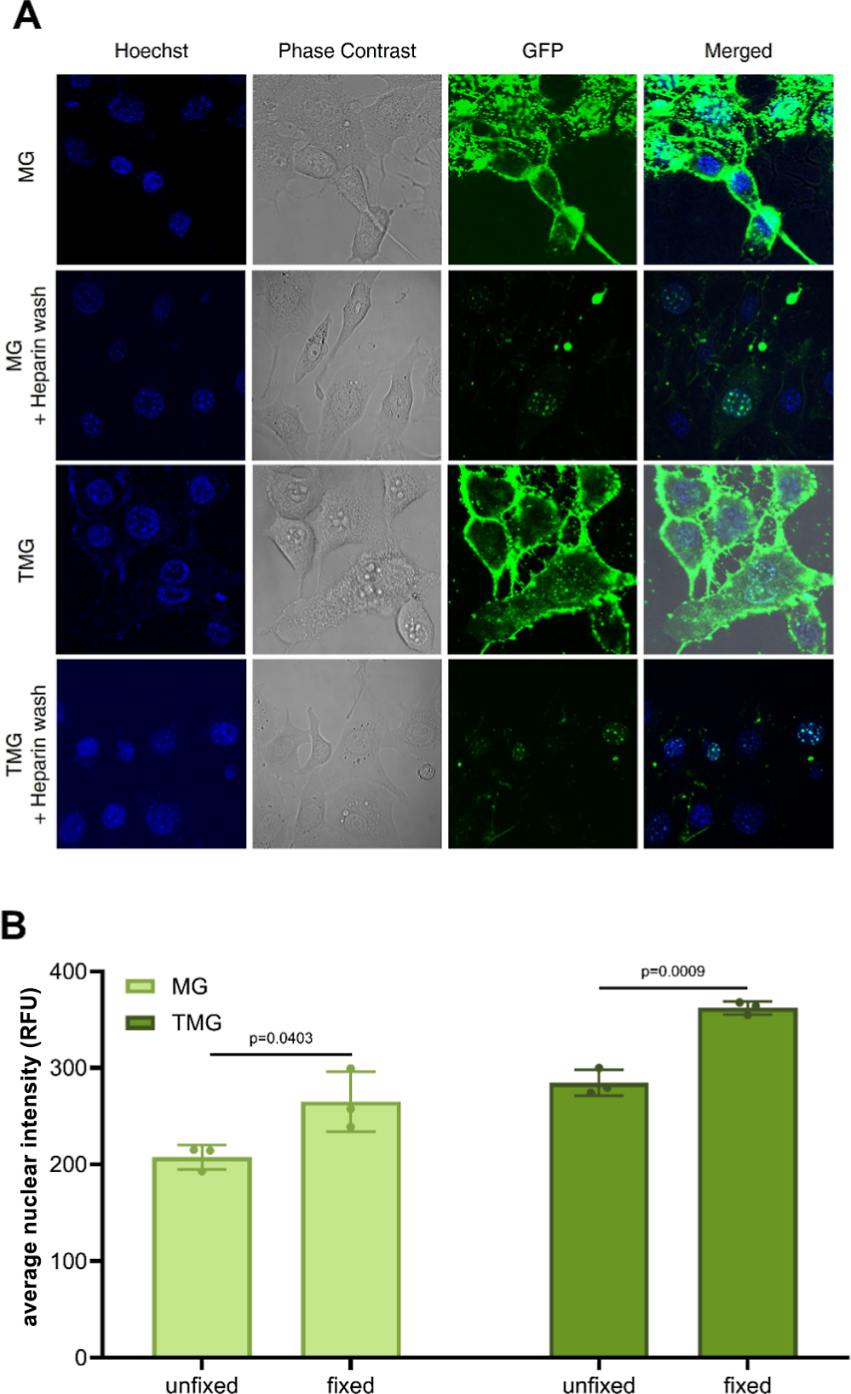
Representative live-cell images showing SP and CPP-FP uptake. (A) Investigation of MG and TMG uptake in the presence and absence of heparin. Live-cell imaging of representative NIH3T3 cells incubated with 4 μM MG and 4 μM TMG without or with a postincubation treatment of 0.5 mg/mL heparin. (B) Effect of cellular fixation on MG and TMG uptake assessed using the ImageXpress Pico system. Average nuclear intensity in relative fluorescent units (RFU) of GFP-positive NIH3T3 cells incubated with 4 μM MG or 4 μM TMG under unfixed versus fixed conditions (*n* = 3 independent experiments; data shown as mean ± SD, paired student’s *t*-test).

In addition to live-cell imaging, fixation techniques, followed by fluorescence microscopy, were previously employed to study protein uptake. However, multiple studies have clearly demonstrated that fixation is not suitable for this purpose, as it is known to skew and significantly overestimate protein internalization levels.^21,39^ MG and TMG uptake into fixed NIH3T3 cells was increased by approximately 25% compared to their nonfixed controls (Figure 3B), further supporting previous observations on the unsuitability of chemical fixation tools when studying CPP-FP/SP uptake.

Once the CPP-FP/SP uptake is ascertained, their internalization levels should be quantified. Numerous tools can be used for this purpose;^16,34^ two such tools are IFC and ImageXpress Pico. The former integrates cell sorting and fluorescence microscopy and allows for discrimination between membrane-bound and incorporated protein, while the latter determines the mean fluorescence intensity from the incubated protein per cell. MG and TMG internalization levels were successfully quantified using both IFC (Figure 4A,B) and ImageXpress Pico (Figure 4C), defining colocalization of recombinant protein signal with heterochromatic foci in NIH3T3 nuclei^36,37^ as the positivity criterion. TMG exhibited superior internalization levels compared to its MG counterpart by approximately 50% (Figure 4B,C), while the minMG negative control displayed negligible uptake (Figure 4B). A higher number of positive nuclei, by an order of magnitude, was observed when data was acquired with the ImageXpress Pico compared to IFC, owing to the ability of the latter to distinguish between unincorporated and internalized signal. This makes IFC, a relatively new application in this field, a powerful approach for accurately evaluating CPP-FP/SP transduction levels.

**Figure 4:**
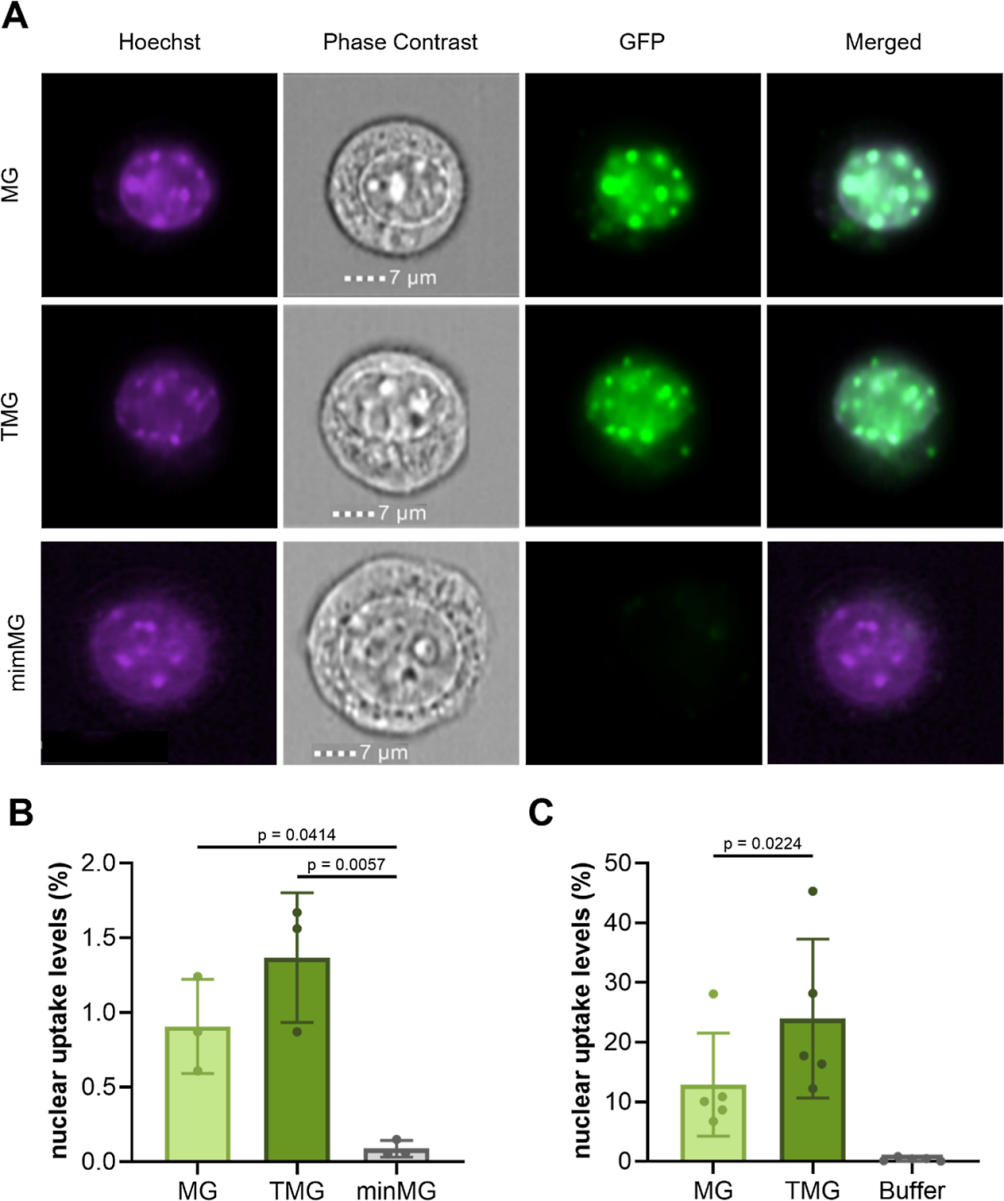
(A) Representative MG, TMG, or minMG (3 μM each)-incubated NIH3T3 cells analyzed by IFC. (B) IFC quantification of TMG, MG, or minMG (3 μM each) nuclear uptake. The data represents mean values ± SDs of three biological replicates; statistical significance was determined using one-way ANOVA. (C) Quantification of cells positive for nuclear recombinant MG and TMG (3 μM each) compared to buffer control using ImageXpress Pico. Statistical significance of the difference in nuclear uptake between MG and TMG was determined using a paired Student’s *t*-test (*p* = 0.0224, *n* = 5). Adapted from ref 24. Available under CC BY 4.0. Copyright 2024 Wiley.

### 3.3 ImageXpress Pico Quantification Provides Insight into CPP-FP/SP Endosomal Trafficking

Endosomes are known traffickers of CPP-FP and SPs.^3,19,40,41^ To investigate their involvement, the ImageXpress Pico can be used to ascertain whether coincubation with compounds known to promote endosomal escape,^40^ such as chloroquine (CHQ) and sucrose (Suc), enhances cellular uptake. Subsequent increases in transduction levels (Figure 5A) clearly point to the endosomal involvement in the internalization process.

**Figure 5:**
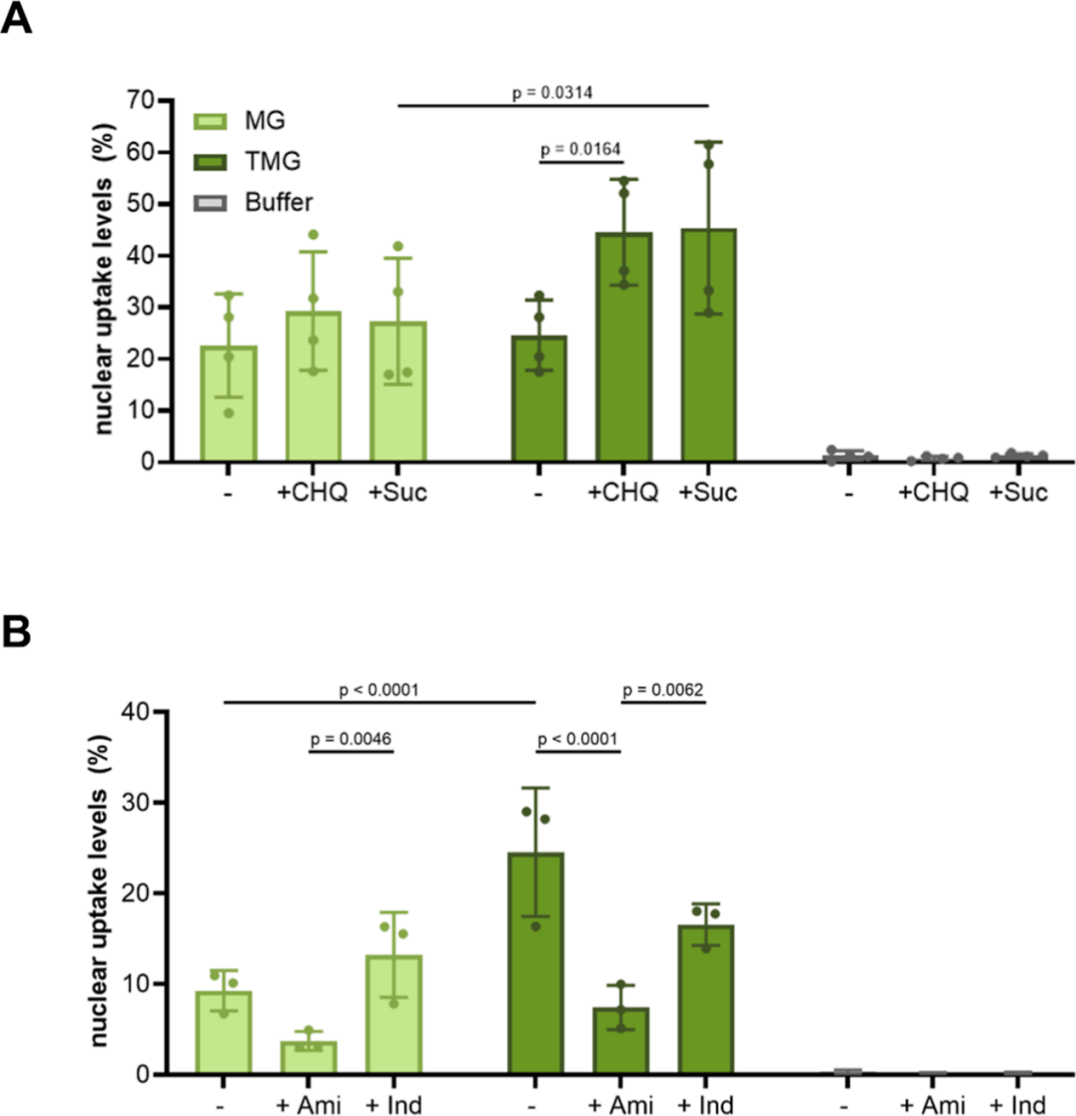
Validation of endosomal involvement in MG and TMG uptake (4 μM each) into NIH3T3 cells. (A) Quantification of MG and TMG uptake in the presence of endosome-disrupting compounds: 100 μM chloroquine (CHQ) or 80 mM sucrose (Suc) versus a buffer control. Data represents mean ± SD of four biological replicates. (B) Quantification of MG and TMG transduction into NIH3T3 cells preincubated with either 0.5 mM amiloride (Ami) or 0.5 mM indomethacin (Ind). The data is presented as the means ± SDs of three biological replicates. For both experiments, statistical significance was determined by two-way ANOVA. Adapted from ref 24. Available under CC BY 4.0. Copyright 2024 Wiley.

To further delve into the exact mechanism of endocytic trafficking, cells designated for transduction with recombinant proteins were preincubated with inhibitors targeting distinct endocytic pathways. One such inhibitor is amiloride, which is known to suppress macropinocytosis,^42^ the most common CPP-FP/SP trafficking route.^3^ The same experiment was also performed in the presence of indometacin, which targets caveolae-mediated endocytosis—another known CPP internalization pathway.^3,43^ Protein transduction levels were then quantified using ImageXpress Pico. Clear decreases in transduction levels like the one observed for both MG and TMG in the presence of amiloride strongly implicate macropinocytosis as their major trafficking route (Figure 5B). Modest declines in recombinant protein uptake such as the one observed for TMG, upon preincubation with indomethacin, may potentially point to caveolae-mediated endocytosis serving as an additional, albeit secondary, endocytosis transport pathway for this protein (Figure 5B). These results demonstrate the utility of ImageXpress Pico not only in quantifying CPP-FP/SP transduction but also in gaining insight into the mechanistic details of their cellular ingress.

### 3.4 SP Sequence Scanning Reveals CPP-like Motifs Critical to SP Uptake

SP transduction is known to be mediated by disordered and positively charged motifs.^3,15,17,19^ Mapping the location and analyzing the contribution of these motifs to SP uptake may prove beneficial in gaining additional insight into the sequence requirements, as well as into the mechanistic aspects of SP internalization. For this purpose, CPPSite 2.0, a software that scans for putative CPP motifs,^32,33^ was first used to search the sequence of interest (here MeCP2) for potential CPP motifs. If such motifs are identified (Figure 6), their contribution to SP transduction is assessed using a combination of computational and experimental work. The computational component involves analyzing previous findings, which may help rule out certain candidates (M1 and M4, marked in gray in Figure 6). For instance, if certain sequences were present in nontransducing variants of the same protein elsewhere,^34^ they can be considered dispensable for SP uptake.^24^ The experimental approach involves assessing the internalization efficiency of the remaining CPP candidates (M2 and M3, marked in light and dark blue, respectively, in Figure 6). Protein constructs containing these CPP sequences tethered to eGFP are recombinantly expressed, purified, and evaluated by live-cell imaging. As the position of the CPP sequence (upstream or downstream of its eGFP fusion partner) can influence internalization efficiency,^44^ both N-terminal and C-terminal fusions should be examined. If any of these protein variants are found to successfully mediate transduction, the internalization of a recombinant SP devoid of this sequence is investigated.^24^ Uptake abrogation would point to the involvement of the missing CPP motif in parent SP transduction (M3, marked in bold in Figure 6).

**Figure 6:**
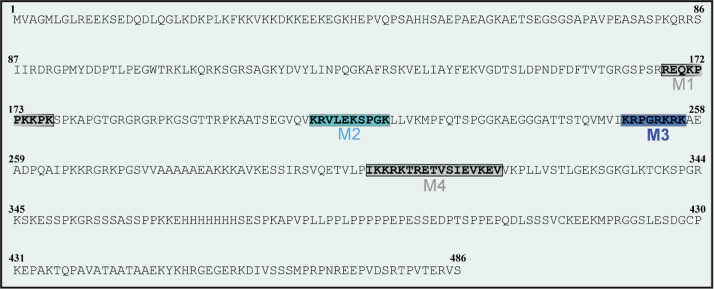
Candidate CPP motifs within a SP (MeCP2) sequence. M1 and M4, the motifs whose involvement can be excluded based on previous work, are marked in gray. M2 and M3, which were tested experimentally, are denoted in light blue and dark blue, respectively. M3, the culprit CPP motif, is also marked in bold. Adapted from ref 24. Available under CC BY 4.0. Copyright 2024 Wiley.

### 3.5 Co-immunoprecipitation Can Serve as a Valuable CPP-FP/SP Functional Validation Tool

Functional experiments are key in assessing the CPP-FP/SP activity in cellulo. CoIP tests the ability of an internalizing protein to recruit known binding partners. In this case, when added to NIH3T3 cells, both MG and TMG have recruited HDAC3 (Figure 7A), a previously described MeCP2 interactor.^45^ All such interactions should be verified by spiking untreated cell lysates with the recombinant proteins in question (Figure 7B). Finally, all nuclear fractions should be tested for potential cytoplasmic contamination, a necessary quality control step validating proper cellular subfractionation (Figure 7C). Taken together, this functional validation tool, used on a variety of occasions,^24,34^ is of great value when ascertaining CPP-FP/SP activity.

**Figure 7:**
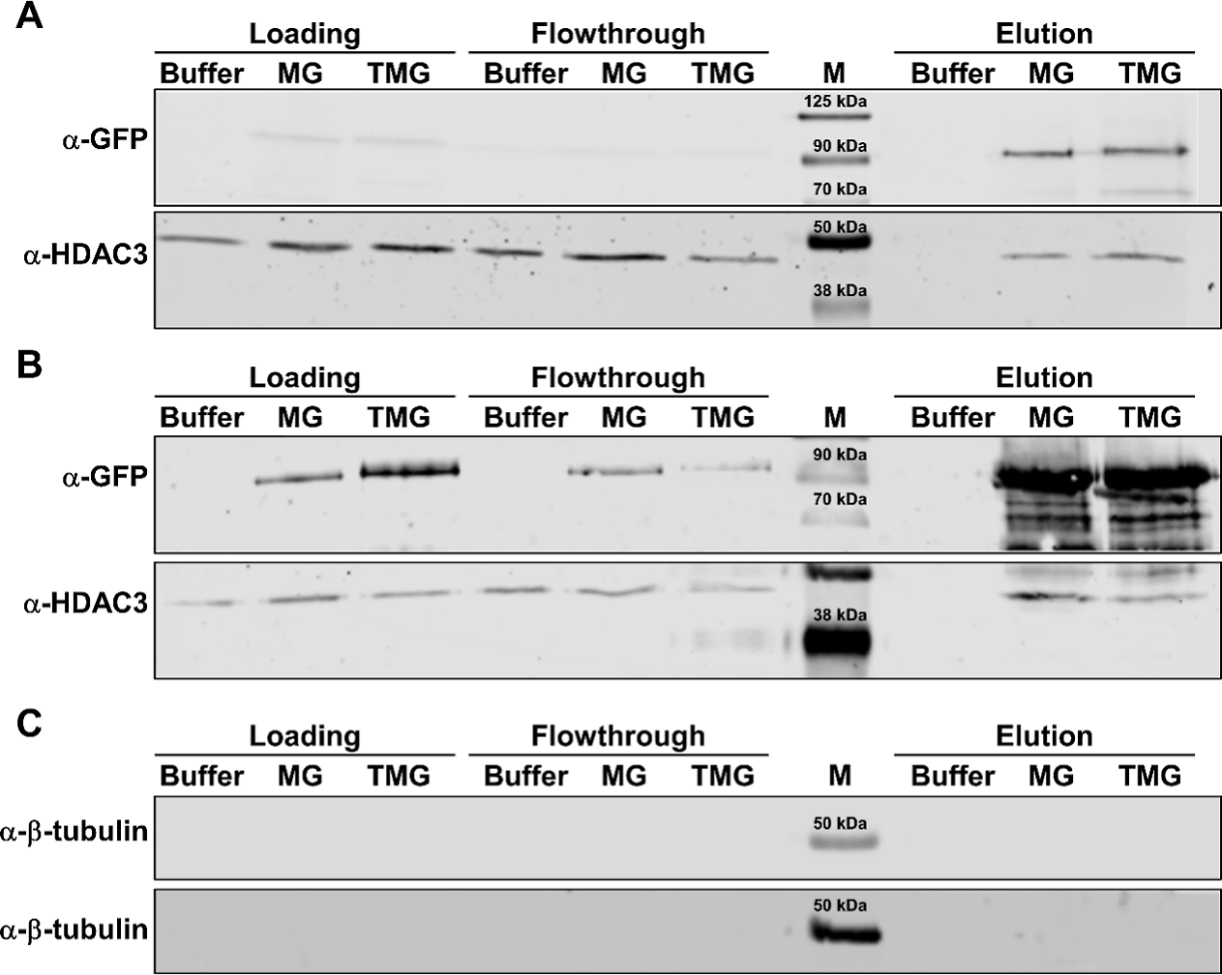
Functional validation of CPP-FP and SP uptake. (A) Nuclear fractions of NIH3T3 cells incubated with storage buffer, MG, or TMG. (B) NIH3T3 nuclear cell lysates spiked with storage buffer, MG, or TMG. Both blots were stained for the presence of eGFP-tethered MeCP2 (anti-GFP antibody) and HDAC3 (anti-HDAC3 antibody). (C) Nuclear extracts of NIH3T3 cells incubated with storage buffer, MG, TMG (top), or lysate-spiked with MG and TMG (bottom), stained for the presence of the cytosolic marker β-tubulin. M—Chameleon DUO prestained marker. Protein concentrations for the CoIP and spike experiments were 1.5 μM and 150 nM, respectively. Adapted from ref 24. Available under CC BY 4.0. Copyright 2024 Wiley.

## 4 Discussion

CPPs and SPs possess cell transduction capabilities, making them valuable tools for intracellular delivery in both research and therapeutic contexts. However, the lack of standardized protocols for their isolation, functional characterization, and uptake investigation has led to significant variability and reproducibility issues across studies. In this work, we present an integrated workflow combining optimized sample preparation, biochemical analysis, and advanced imaging techniques to enable robust CPP-FP and SP characterization.

Plasmid construct design, as well as the optimization of protein expression, are crucial components of a successful recombinant protein production pipeline.^22^ When expression conditions are established, the downstream purification protocol should be considered. CPP-FPs and SPs are commonly captured under denaturing conditions with subsequent refolding on an affinity column.^16,46^ CPP-FPs in a denatured state are occasionally employed in uptake studies with the expectation that they will be refolded by cellular chaperones.^47,48^ However, this approach can lead to several complications. Denatured proteins may interact nonspecifically with the cell membrane due to exposed hydrophobic regions, leading to diminished uptake. Furthermore, not all proteins refold efficiently *in vitro* or *in cellulo* and may assume a conformation that differs from their native state.^49–51^ Low levels of correctly folded protein may compromise performance in internalization, binding, and functional assays. To avoid these issues, we purified our proteins under native conditions. This approach yields a stable and properly folded protein suitable for downstream uptake and functional studies.

Protein stability and aggregation propensity have a direct impact on cellular uptake efficiency and, consequently, on subsequent readouts.^48^ Hence, structural characterization of purified CPP-FPs/SPs serves as an essential intermediate step before any downstream applications. Circular dichroism (CD), a technique commonly used to probe secondary structure, was previously employed to characterize several CPP-FPs and SPs.^52,53^ Nuclear magnetic resonance (NMR) spectroscopy provides insight into structured and disordered protein regions^23,54–56^ and can also serve as a validation tool to compare folds following native and denaturing purifications.^57^ However, CD is hampered by a low sensitivity to aggregate formation and poor suitability for long-term measurements, while NMR is limited by spectral overlap linked to molecular size as well as by prohibitive (milligram) amounts of sample required for analysis. In contrast, DLS efficiently detects high-molecular-weight species and allows buffer screening in a high-throughput format over extended time periods. We identified buffer conditions in which the protein constructs maintained solubility and resisted aggregation for 72 h, exceeding typical incubation periods used in cellular uptake studies.

Live-cell conditions are essential for accurately assessing protein uptake and localization. Cellular fixation can artificially permeabilize membranes and disrupt endosomes, leading to misinterpretation and overestimation of internalization efficiency and compartmentalization.^21,39^ We therefore relied on live-cell fluorescence microscopy and IFC to quantify protein uptake in native cellular contexts. IFC, in particular, combines high-throughput analysis with single-cell resolution, enabling precise quantification of intracellular localization and colocalization events.^28–30^ This technology overcomes limitations of conventional flow cytometry, which often overestimates uptake due to its inability to distinguish between surface-bound and internalized protein, especially with isolated cell nuclei.^15,16,30,39^ In our study, both MG and TMG were successfully internalized into NIH3T3 cells, with TMG showing ∼50% higher uptake. These findings were consistent across ImageXpress Pico and IFC data, illustrating the value of using multiple orthogonal readouts for validation.

Notably, we observed markedly higher uptake levels of both MG and TMG when measured using the ImageXpress Pico system compared to those observed by IFC. This discrepancy likely reflects fundamental differences in detection sensitivity and signal interpretation between the two platforms. While imaging-based plate readers quantify total cellular fluorescence, they cannot reliably distinguish between surface-bound and internalized protein. In contrast, IFC provides single-cell resolution and spatial context, enabling more reliable discrimination between membrane-associated and truly intracellular fluorescence. Thus, IFC yields a more conservative yet also a more accurate estimation of protein internalization under live-cell conditions. These findings support the considerable value of IFC in accurately probing and quantifying CPP-FP/SP cellular internalization at single-cell resolution.

The use of an appropriate noninternalizing negative control is strongly encouraged. Ideally, such a control should possess properties similar to those of its internalizing counterpart. Here, a truncated protein variant of MeCP2, minMeCP2,^58^ fused to eGFP, which was deemed transduction-incompetent in another study,^24^ was employed. Alternatively, nontransducing fluorescent fusion protein constructs such as eGFP^11,13^ or mCherry^15,16^ can also be utilized.

To determine whether endocytosis plays a role in CPP-FP/SP transduction, we have conducted co- or preincubation experiments of our protein constructs with endosome-disrupting or endocytosis-inhibiting compounds, respectively, with subsequent numerical uptake evaluation. While these experiments were carried out using the ImageXpress Pico, similar experiments can also be conducted using IFC as both tools can quantify cellular uptake. Our data point to endocytosis, particularly macropinocytosis, serving as the main MeCP2-derived CPP-FP/SP ingress route under physiological conditions.^3^ This was validated through coincubation with compounds promoting endosomal escape (chloroquine and sucrose)^24,40^ and through the use of selective endocytosis inhibitors (amiloride and indomethacin),^42,43^ respectively. To target endocytic pathways, siRNA-mediated knockdown can also be used, albeit with increased experimental complexity.^34,59^ These results support previous findings, highlighting macropinocytosis as the chief CPP internalization route.^3,4^

To identify transduction-relevant motifs within SP sequences, we utilized the CPPSite 2.0 tool to locate CPP-like regions. The candidate sequences, which could not be ruled out based on previous work, were cloned into eGFP fusion proteins and tested for uptake. Protein constructs showing internalization ability served as the basis for the design of the appropriate SPΔCPP mutants for loss-of-function validation experiments. An abolition in uptake supports the functional role of the deleted motif. The candidate CPPs in MeCP2 were found to be rich in arginine, lysine, and proline—three residues with a demonstrated role in CPP transduction.^3^ In addition, these sequences exhibited a sequence homology to CPPs from various viral proteins. Notably, the sequence mediating MG uptake displays a similarity to TAT.^24^ These findings point to an apparent link between SPs and CPPs. The presence of protein transducing motifs appears to confer on SPs not only their uptake capability but also a specific mechanistic mode of entry.

While the identification of CPP candidate motifs to gain insight into the determinants of cellular ingress is of importance, additional sequence and structural elements can also modulate transduction levels. A higher abundance of arginine and lysine has been shown to enhance uptake by increasing interactions with heparan sulfates on the cell surface and trigger actin remodeling.^2,60^ The presence of aromatic residues raises cellular incorporation levels through increased intercalation into the cell membrane^61^ and more efficient endosomal escape.^62,63^ Furthermore, protein secondary structure is known to play a significant role, as α-helical elements have been reported to confer increased CPP-FP internalization activity.^4^ These findings have inspired the design of “secondary CPPs”—initially unstructured motifs which assume helical structures in amphipathic environments.^64–66^

Functional validation of transducing CPP-FPs or SPs is key to ascertaining their downstream activity. Here, both MG and TMG successfully coprecipitated HDAC3, a well-described MeCP2 binding partner,^34,45^ indicating that their intracellular levels were sufficient to engage in protein–protein interactions. Beyond CoIP, the functionality of CPP-FPs and SPs can be probed in *in vitro* binding assays, reporter constructs for cytosolic processing, or nuclear recombination experiments.^15,16,19^ Fluorescent tags may also be replaced with alternative labels for uptake quantification.^34^ Finally, tracking post-translational modifications *in cellulo* can provide a more rigorous insight into CPP-FP/SP activity following delivery.^34,67^

This work has a number of limitations. First, derivatives of only one protein were used. Other work has shown that live-cell imaging as well as additional methods presented here are broadly applicable to other CPP-FPs and SPs.^11,19^ In addition, the cellular model (NIH3T3 fibroblasts) is not phenotypically relevant for therapeutic applications. This cell line was chosen due to the ability of MeCP2 to colocalize at the heterochromatic foci of NIH3T3 cells,^36,37^ which facilitates efficient uptake quantification. However, other cell models are compatible with the experimental approaches outlined in the study.

The application of the aforementioned protocol should result in higher reproducibility in CPP-FP/SP studies. The combination of increasing study fidelity with emerging improvements in efficiency of protein uptake^18^ should pave the way to wider use of these delivery systems to target various disorders as alternatives to or in concert with gene therapy.

## 5 Conclusions

In summary, this study provides a comprehensive and standardized workflow for the production, characterization, and functional analysis of CPP-FP and SP using TMG and MG constructs as proof of concept. It highlights the importance of proper sample preparation and biochemical characterization, an integral requirement before proceeding to downstream internalization experiments. The value of conservative, nondisruptive live-cell techniques in adequately assessing CPP-FP and SP transduction, as well as its quantitative and mechanistic aspects, is emphasized. The apparent link between SPs and CPPs is also examined. Finally, additional tools to study functional applications of CPP-FP/SP internalization are described. These methodologies, coupled with recent developments in protein isolation and cellular imaging, should spur further advances in the field of protein-based therapy and accelerate the development of next-generation biologics.

## Supplementary Material





## Notes

Representative z-stack scans (MP4)
Additional representative live-cell images; list of specialized reagents and solutions; detailed protocol for protein isolation, characterization, uptake analysis, sequence mapping, and functional validation; and representative DLS buffer screen (PDF)

## Acronyms

CPP: cell-penetrating peptides
CPP-FP: CPP-fusion proteins
SP: supercharged protein
DLS: dynamic light scattering
MeCP2: methyl-CpG-binding protein 2
TAT: transactivator of transcription
MG: MeCP2-eGFP
TMG: TAT-MeCP2-eGFP
CoIP: co-immunoprecipitation
LPS: lipopolysaccharides
MTT: 3-[4,5-dimethylthiazol-2-yl]-2,5 diphenyl tetrazolium bromide
CHAPS: 3-[(3-cholamidopropyl)­dimethylammonio]-1-propanesulfonate
CYMAL-5: 5-cyclohexyl-1-pentyl-β-d-maltoside
NTM: *n*-nonyl-β-d-thiomaltoside
IFC: imaging flow cytometry
HDAC3: histone deacetylase 3
CHQ: chloroquine
Suc: sucrose
NMR: nuclear magnetic resonance
HSQC: heteronuclear single quantum coherence
DMEM: Dulbecco’s modified Eagle’s medium
IPTG: isopropyl β-d-1-thiogalactopyranoside
PEG 400: polyethylene glycol 400
DTT: dithiothreitol
PIC: protease inhibitor cocktail
PI-His: protease inhibitor cocktail-His
BME: β-mercaptoethanol
SDS: sodium dodecyl sulfate
