# Supplementary material for: Cell-Penetrating Peptides and Supercharged Proteins: A Comprehensive Protocol from Isolation to Cellular Uptake
Source: Mol Pharm. 2026 Feb 19;23(3):1845–57. doi: 10.1021/acs.molpharmaceut.5c01560 (PMC12958280; doi:10.1021/acs.molpharmaceut.5c01560)
Supplement: Supplementary file 2 [file mp5c01560_si_002.pdf]

Cell-Penetrating Peptides and Supercharged Proteins: A Comprehensive Protocol from Isolation to Cellular Uptake

Alexander V. Beribisky[1], Victoria Sarne[1,2], Anna Huber[1], Markus Hengstschläger[1], Franco Laccone[1] and Hannes Steinkellner*[1]

[1]Institute of Medical Genetics, Center for Pathobiochemistry and Genetics, Medical University of Vienna, Währinger Straβe 10, 1090, Vienna, Austria

[2]Vienna Doctoral School of Pharmaceutical, Nutritional and Sport Sciences (PhaNuSpo), University of Vienna, Josef-Holaubek-Platz 2, 1090, Vienna, Austria

Supplementary Information

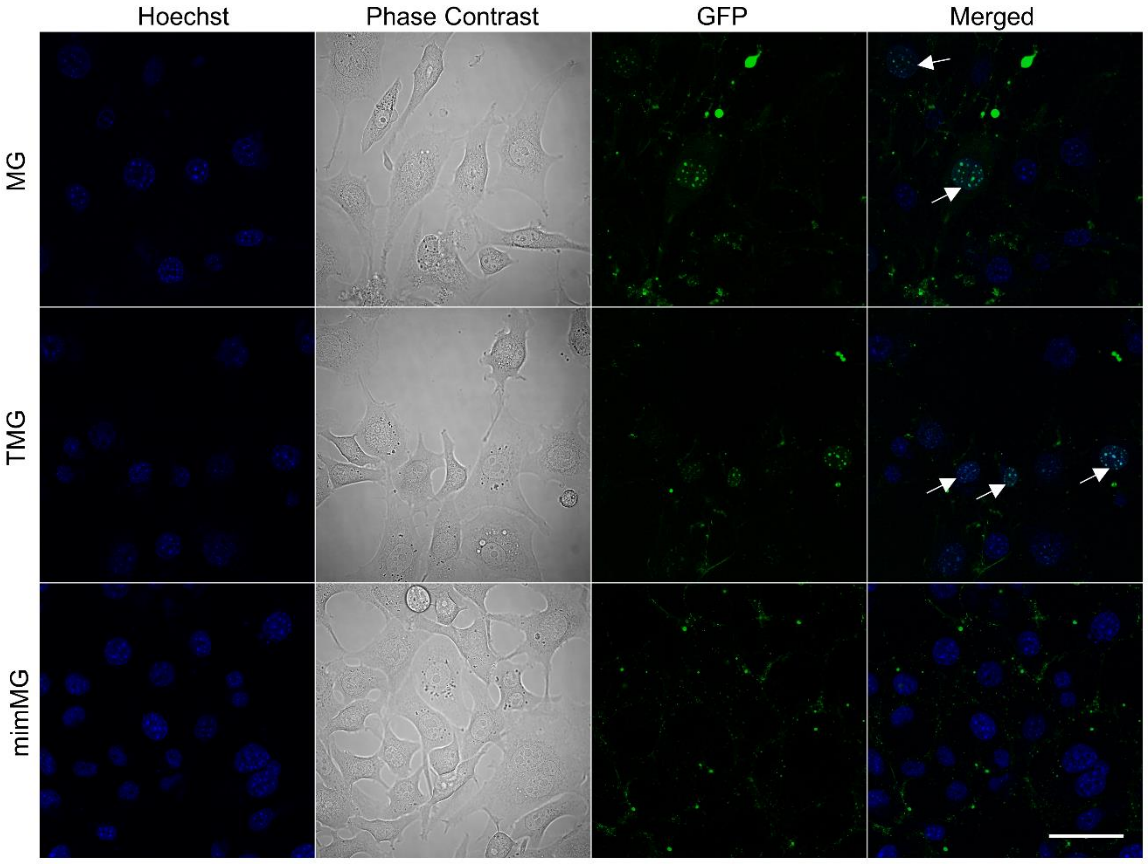

Figure S1. Initial assessment of SP and CPP-FP uptake capability. Representative live-cell images of NIH3T3 cells incubated with MG, TMG and a minMG negative control (4 μM each). Arrows indicate sample-positive cells showing co-localization with nuclear foci. Scale bar = 50 μm.

Table S1. List of specialized reagents used in this study, along with their catalogue numbers.

Name	Manufacturer, Catalogue number
E. cloni EXPRESS BL21(DE3) Electrocompetent Cells	Biocat, #60300-1-LU
Strep-Tactin XT column	IBA, #2-5014-001
Biotin	IBA, #2-1016-002
Disposable PD-10 desalting column	Cytiva, #17085101
Amicon® Ultra Centrifuge filter, 10 kDa MWCO	Sigma, #UFC90100
HiLoad 16/60 S-200 gel filtration column	Cytiva, #28989335
Precision Plus Protein™ Dual Xtra Prestained Protein Standards	Bio-Rad, #1610377
Dulbecco's modified Eagle's medium (DMEM)	Gibco, #41966
10% fetal bovine serum	Sigma, #F9665
1% penicillin–streptomycin	Gibco, #15140122
96-well plate	Corning, #353075
8-well collagen IV-treated μ-slides	Ibidi, #80822
1.5 μM Hoechst 33342	Thermo Scientific, #62249
Live cell imaging solution	Thermo Fisher Scientific, #A14291DJ
Protease Inhibitor Cocktail (PIC)	Sigma, #P8340
Protease Inhibitor Cocktail-His (PI-His)	Sigma, #P8849
S220 Focused ultrasonicator	Covaris, #500217
Bio-Rad protein assay	Bio-Rad, #5000006
MagStrep"type 3"XT beads	IBA, #2-4090-002
4x Laemmli Sample Buffer	Bio-Rad. #1610747
Chameleon Duo pre-stained marker	LI-COR, #928-60000
iBlot® 2 Gel Transfer Device	Invitrogen, #IB21001
Intercept® Blocking Buffer	LI-COR, #927-60001
Intercept® Antibody Diluent	LI-COR, #927-65001

anti-GFP antibody	Abcam, #ab290
anti-MeCP2 antibody	Sigma, #SAB1404063
anti-HDAC3 antibody	CST, #85057S
anti-β-tubulin antibody	Sigma, #T4026
IRDye® 680RD Goat anti-Mouse IgG Secondary Antibody	LI-COR, #926-68070
IRDye® 800CW Goat anti-Mouse IgG Secondary Antibody	LI-COR, #926-32211

Table S2. Composition of solutions prepared and used in this study.

Solution name	Composition
LB solution	10 g/L Trypton, 5 g/L yeast extract, 10 g/L NaCl
LB rich	LB solution + 2.5 g/L meat extract
Lysis buffer	100 mM Tris–HCl, 500 mM NaCl, 250 mM Urea, 1 mM EDTA, 0.1% (v/v) Triton X-100, 0.1% (v/v) Tween 20, 8% (v/v) Glycerol, pH = 8.0
Lysis buffer additives	5 mM β-mercaptoethanol (BME), 50 μg/mL lysozyme, 500 U benzonase, 100 μg/mL phenylmethylsulfonyl fluoride, 500 μL PI-His
Equilibration buffer	Lysis buffer + 5 mM BME
Wash buffer	Lysis buffer + 1.5 M NaCl + 5 mM BME
Elution buffer	Lysis buffer + 50 mM Biotin + 5 mM BME
Gel filtration chromatography buffer	DPBS, 300 mM NaCl, 1 mM Dithiothreitol (DTT), 10% (v/v) Glycerol, 5% Isopropanol, 0.02% (w/v) NaN_3, pH = 7.2
Storage buffer base	DPBS, 200 mM NaCl, 10% (v/v) Glycerol, pH = 7.2
Storage buffer with CHAPS	Storage buffer + 0.05% (w/v) 3-[(3-Cholamidopropyl)dimethylammonio]-1-propanesulfonate (CHAPS)
NP-40 buffer	0.1% NP-40, 1X PIC, 1 mM DTT in PBS
CoIP lysis buffer	20 mM HEPES, 10 mM KCl, 1.5 mM $MgCl_2$, pH = 7.6

Table S3. Protocol for CPP-FPs and SPs expression in *E. coli*. Steps specific to this study are indicated in brackets.

Stage 1 – Transformation
1) Thaw BL21(DE3) *E. coli* on ice.

2) Add 1 µL DNA sample to the 50 µL of cells on ice; stir gently with pipette tip.
3) Carefully pipette 50 µL of the mixture into an electroporation cuvette.
4) Gently tap the cuvette to remove air bubbles.
5) Transform the plasmid into the cells by either electroporation (25 µF, 200 Ω, 2500 V) or by heat shock (42 °C for 30 sec and leave on ice for 5 min).
6) Add 950 µL of expression recovery medium or LB immediately.
7) Transfer to a sterile clean microcentrifuge tube.
8) Incubate at 37 °C with shaking (300 rpm) for 1 hour.
9) Plate 2-5 µL on LB plates with the appropriate antibiotic marker (Kan, 50 µg/mL).
10) Incubate at 37 °C overnight.
Stage 2 – Pre-culture inoculation
1) Store plates at 4 °C in the morning.
11) In the afternoon, inoculate single colonies into 10 mL LB with the appropriate antibiotic marker (Kan, 50 µg/mL).
2) Incubate overnight (~16 hours) at 37 °C with shaking at 250 rpm.
Stage 3 – Main culture growth and induction
1) Inoculate 250 mL of LB-rich medium (with 50 µg/mL kanamycin) with a 1:50 dilution of overnight culture.
2) Grow at 37 °C with shaking at 250 rpm until OD_{600} reaches 0.8.
3) If expressing at 20 °C, cool cultures at 4 °C for 30 min.
4) Induce expression with Isopropyl β-d-1-thiogalactopyranoside (IPTG, 1 mM).
5) Incubate as appropriate (20 °C for 20 hours with shaking at 250 rpm).
**Critical note:** OD_{600} at induction, IPTG concentration, incubation temperature, and duration should be carefully optimized for each construct to achieve maximal soluble protein yield.
Stage 4 – Cell harvesting
1) Harvest the cells by centrifugation at 4,200 × *g* for 30 min at 4 °C.
2) Store pellets at -80 °C until further use.

Table S4. Protocol for purification of CPP-FPs and SPs. Steps specific to this study are indicated in brackets. Critical notes are denoted in bold.

Stage 1 – Cell lysis and clarification

1) Thaw bacterial pellets (from a 250 mL LB-rich culture) on ice.

2) Re-suspend in 50 mL lysis buffer with additives, incubate on ice for 15 min.

3) Sonicate 6x 45 sec with a cooling interval of 2 min to avoid excessive heating.

4) Centrifuge lysate at 15,000 rpm (Sorval fixed-angle rotor) for 45 min at 4 °C.

5) Filter supernatant through a 0.45 μm filter.

Stage 2 – Strep-Tactin®XT affinity chromatography

1) Pre-equilibrate the Strep-Tactin XT column with 20 mL equilibration buffer.

2) Load filtered lysate onto the column, collect flowthrough in a 50 mL tube.

3) Treat the column-bound protein with 50 mL wash buffer.

4) Add 15 mL elution buffer; collect between 7.5 mL and 12.5 mL.

Critical note: Use high-salt wash buffer for DNA/RNA-binding proteins to remove nucleic acids, which may interfere with downstream functionality.

Stage 3 – First buffer exchange and concentration

1) Exchange eluted protein into the gel filtration buffer using a PD-10 column.

2) Concentrate to 2 mL using an MWCO spin concentrator (10,000 MWCO, 2,000 × *g*, 2-3.5 hours, at 4 °C).

Critical note: Do not concentrate to volumes below 2 mL, this may result in sample precipitation and/or aggregation.

Stage 4 – Gel filtration chromatography

1) Pre-equilibrate HiLoad 16/60 S-200 column 24 hours in advance with one column volume (CV) of ddH_2O and 1 CV of filtered, degassed gel filtration column buffer.

2) Centrifuge sample (10,000 × *g* for 3 min, at 4 °C).

3) Load onto column and initiate run program.

4) Collect protein at the target elution volume.

5) Re-equilibrate column with 1 CV each of ddH_2O and 20% ethanol.

Critical note: Buffer composition, flow rate and protein elution volume should be optimized before carrying out large-scale purifications.

Stage 5 – Second buffer exchange and final concentration

1) Pool peak fractions and exchange into storage buffer using PD-10 column.

2) Concentrate to 1.5-2 mL using a MWCO concentrator (10,000 MWCO, 2,000 × *g*, 1-2 hours, at 4 °C).

3) Centrifuge at 10,000 × *g* for 3 min, at 4 °C.

Critical Note: Do not reduce volume below 1 mL to avoid potential protein aggregation.

Stage 6 – Second buffer exchange and final concentration

1) Add Triton X-114 to 2.5% (v/v), vortex until fully dissolved and solution turns turbid.
2) Incubate 30 min on ice, then store 10 min at 37 °C, without shaking.
3) Centrifuge at 16,000 × *g* for 15 min at 25 °C.
4) If phase separation is incomplete, add 200 μL storage buffer base, vortex until fully dissolved and solution turns turbid.
5) Carefully collect the top, aqueous layer into a fresh microcentrifuge tube.
6) Repeat extraction twice.
7) Remove residual Triton X-114 using a detergent removal column.

Critical note: Proper vortexing and temperature cycling are essential for phase separation and effective LPS removal.

Stage 7 – Final processing and storage

1) Centrifuge the sample at 10,000 × *g* for 3 min, at 4 °C.

   (Add CHAPS to a final amount of 0.05%)
2) Measure protein concentration; adjust to 3.5-4.5 mg/mL.
3) Aliquot as needed.
4) Flash-freeze and store at -80 °C.

Stage 8 – Western blotting

1) Load samples on SDS-PAGE gel (10% acrylamide), run as appropriate (80 V, 2 hours).
2) Blot as appropriate (iBlot® 2 Gel Transfer Device, program 0, 7 minutes).
3) Block the membranes (10 mL of Intercept® Blocking Buffer, 1 hour).
4) Add primary antibodies (anti-GFP (1:1000), anti-MeCP2 (1:1000), in 5 mL Intercept® Antibody Diluent).
5) Incubate overnight with rotation at 4 °C.
6) Add secondary antibodies (1:20,000 in 5 mL Intercept® Antibody Diluent).
7) Incubate for 1 hour under gentle agitation at 4 °C.
8) Image the blots (Odyssey CLx imaging device).
9) Analyze (ImageStudio software, LI-COR Biosciences).

Table S5. A TMG DLS buffer screen with intensity and radii ratios of final (one week at 25 °C) to initial measurements denoted. Values closer to 1 have a green background in the table heatmap. The buffer conditions (A1) used for subsequent experiments are marked in bold. Percentages of glycerol and polyethylene glycol 400 (PEG 400) are specified in v/v, detergent percentages are in w/v. Adapted from (1). Available under CC BY 4.0. Copyright 2022 Springer Nature Link.

	1	2	3	4	5	6	7	8	9
A	**DPBS pH=7.2**	DPBS pH=7.2	DPBS pH=7.2	DPBS pH=7.2	DPBS pH=7.2	DPBS pH=7.2	DPBS pH=7.2	DPBS pH=7.2	DPBS pH=7.2
	**10% Glycerol**	10% Glycerol	10% Glycerol	10% Glycerol	10% Glycerol	10% Glycerol	10% Glycerol	10% Glycerol	10% Glycerol
	**200 mM NaCl**	400 mM NaCl	200 mM NaCl	400 mM NaCl	200 mM KCl	400 mM NaCl	200 mM KCl	400 mM KCl	400 mM NaCl
	**0.05% CHAPS**	0.05% CHAPS	1 mM DTT	1 mM DTT	0.05% CHAPS	0.05% CHAPS	1 mM DTT	1 mM DTT	1 mM DTT
			0.05% CHAPS	0.05% CHAPS			0.05% CHAPS	0.05% CHAPS	0.05% PEG400
									0.05% CHAPS
Intensity	**0.81**	0.9	0.81	1.01	0.89	0.88	0.9	1.09	0.96
Radius	**0.91**	1.64	0.69	0.38	0.89	0.64	0.65	0.79	0.61
B	DPBS pH=7.2	DPBS pH=7.2	DPBS pH=7.2	DPBS pH=7.2	DPBS pH=7.2	DPBS pH=7.2	DPBS pH=7.2	DPBS pH=7.2	DPBS pH=7.2
	10% Glycerol	10% Glycerol	10% Glycerol	10% Glycerol	10% Glycerol	10% Glycerol	10% Glycerol	10% Glycerol	10% Glycerol
	200 mM NaCl	400 mM NaCl	200 mM NaCl	400 mM NaCl	200 mM KCl	400 mM NaCl	200 mM KCl	400 mM KCl	400 mM NaCl
	0.01% CYMAL®-5	0.01% CYMAL®-5	1 mM DTT	1 mM DTT	0.01% CYMAL®-5	0.01% CYMAL®-5	1 mM DTT	1 mM DTT	1 mM DTT
			0.01% CYMAL®-5	0.01% CYMAL®-5			0.01% CYMAL®-5	0.01% CYMAL®-5	0.05% PEG400
									0.01% CYMAL®-5
Intensity	0.83	0.8	0.9	1	0.96	1.01	0.83	0.99	0.94
Radius	0.82	0.47	1.36	0.47	0.66	1.01	0.87	1.33	1.14
C	DPBS pH=7.2	DPBS pH=7.2	DPBS pH=7.2	DPBS pH=7.2	DPBS pH=7.2	DPBS pH=7.2	DPBS pH=7.2	DPBS pH=7.2	DPBS pH=7.2
	10% Glycerol	10% Glycerol	10% Glycerol	10% Glycerol	10% Glycerol	10% Glycerol	10% Glycerol	10% Glycerol	10% Glycerol
	200 mM NaCl	400 mM NaCl	200 mM NaCl	400 mM NaCl	200 mM KCl	400 mM NaCl	200 mM KCl	400 mM KCl	400 mM NaCl
	0.01% NTM	0.01% NTM	1 mM DTT	1 mM DTT	0.01% NTM	0.01% NTM	1 mM DTT	1 mM DTT	1 mM DTT
			0.01% NTM	0.01% NTM			0.01% NTM	0.01% NTM	0.05% PEG400
									0.01% NTM
Intensity	0.6	1	0.91	1	0.92	0.87	0.78	0.9	1.17
Radius	0.55	0.73	0.65	0.8	0.49	0.6	1.16	0.8	1.24
Abbreviations	3-[(3-cholamidopropyl)dimethylammonio]-1-propanesulfonate (CHAPS), 5-Cyclohexyl-1-Pentyl-β-D-Maltoside (CYMAL ®-5), n-Nonyl-β-D-Thiomaltoside (NTM), polyethylene glycol 400 (PEG400)								

Table S6. Protocol for live-cell imaging of CPP-FPs and SPs. Notes specific to this study are indicated in brackets.

Critical notes are denoted in bold.

Stage 1 – Seeding day protocol
1) Seed cells (25,000 NIH3T3 per well) in 8-well µ-slides pre-coated with collagen IV.<br>2) Incubate the cells overnight at 37 °C under a humidified atmosphere containing 5% CO_2, using DMEM supplemented with 10% FBS and 1% penicillin–streptomycin.
**Critical Note:** Optimal seeding density is essential. For NIH3T3 cells, a confluency of approximately 60-70% the following day yields best results for live-cell imaging. Avoid both overly sparse and overly dense cultures to ensure reproducible uptake conditions.
Stage 2 – Imaging day protocol
1) Remove the culture medium.<br>2) Optional: If co-incubations with endocytosis inhibitors are planned, pre-incubate the cells with the appropriate compound for 1 hour.<br>3) Add recombinant protein at the desired concentrations (4 µM), diluted in cell culture medium.<br>4) Optional: For experiments involving endosome-disrupting agents, add them concurrently with the recombinant protein.<br>5) Incubate the protein-treated cells for the desired duration (1 hour).<br>6) Add 1.5 µM Hoechst 33342 for 5 min to stain nuclei.<br>7) Wash cells three times with DPBS containing 0.5 mg/mL heparin.<br>8) Wash cells twice with DPBS.<br>9) Incubate cells with 300 µL live-cell imaging solution.<br>10) Image the cells using a confocal microscope (Leica TCS SP8) under live-cell conditions at 37 °C with 5% CO_2.
**Critical note:** Perform imaging immediately after the final wash to minimize cell stress or death as well as to avoid artifactual protein uptake. Image as few wells as necessary to reduce overall imaging time and hence preserve optimal live-cell conditions.

Table S7. Protocol for IFC of CPP-FPs and SPs. Notes specific to this study are indicated in brackets. Critical notes are denoted in bold.

Stage 1 – Seeding day protocol
1) Seed cells (5x10^5 NIH3T3 per well) in 6-well plates.

2) Incubate the cells overnight at 37 °C under a humidified atmosphere containing 5% CO_2, in the appropriate growth medium (DMEM supplemented with 10% FBS and 1% penicillin–streptomycin).

Critical Note: Optimal seeding density is essential. For NIH3T3 cells, a confluency of approximately 70-80% the following day yields best results for IFC. Avoid both overly sparse and overly dense cultures to ensure reproducible uptake conditions.

Stage 2 – Imaging day, sample incubation

1) Remove the culture medium.
2) Optional: If co-incubation with endocytosis inhibitors planned, pre-incubate the cells with the appropriate compound for 1 hour.
3) Add recombinant protein at the desired concentrations (4 μM).
4) Optional: For experiments involving endosome-disrupting agents, add them concurrently with the recombinant protein.
5) Incubate the protein-treated cells for the desired duration (1 hour).
6) Add 1 μM Hoechst 33342 for 5 min to stain nuclei.
7) Wash cells twice with DPBS.
8) Wash cells three times with DPBS containing 0.5 mg/mL heparin.
9) Wash cells twice with DPBS.
10) Add 0.05% (v/v) trypsin/EDTA for approximately 1 min to detach the cells.
11) Verify detachment under a light microscope.
12) Add media containing 10% FBS to the cells to inhibit trypsinization.
13) Collect the cells in a total volume of 1 mL.
14) Centrifuge the cells at 300 × *g* for 5 min at RT and discard the supernatant.
15) Wash the cell pellet twice with 600 μL of ice-cold DPBS with subsequent centrifugation at 500 × *g* for 5 min at 4 °C.
16) Re-suspend the cell pellet in 70 μL DPBS and store on ice.

Critical Note: Do not exceed the recommended trypsinization time to prevent trypsin-induced increase of membrane permeability and consequently, artificial enhancement of protein uptake.

Stage 3 – Imaging day, data acquisition and processing

1) Acquire data as soon as possible on an imaging flow cytometer (ImageStream®X Mark II).

2) Use the following acquisition channels: Brightfield, a channel to detect the nuclear dye (405 nm, Hoechst) and another to observe CPP-FP/SP signal (488 nm, GFP).
**Critical Note:** Image as quickly as possible to obtain an accurate quantification of CPP-FP/SP internalization which would otherwise be skewed by nuclear dye diffusion as well as by decreases in cell viability over time.

Table S8. Protocol for uptake quantification of CPP-FPs and SPs using ImageXpress® Pico. Notes specifc to this study are indicated in brackets. Critical notes are denoted in bold.

Stage 1 – Seeding day protocol
1) Seed cells (1.3×10^4 NIH3T3 per well) in a clear 96-well plate.<br>2) Incubate overnight at 37 °C in a humidified atmosphere containing 5% CO_2, using DMEM supplemented with 10% FBS and 1% penicillin–streptomycin.<br>3) The optimal cell confluency on the day on experiment day should be approximately 80-90%.
Stage 2 – Imaging day protocol
1) Remove the culture medium.<br>2) Optional: If studies with endocytosis inhibitors are planned, pre-incubate the cells with the appropriate compound (0.5 mM amiloride/0.5 mM indomethacin) prior to protein addition (1 hour).<br>3) Add recombinant protein at the desired concentration (4 μM).<br>4) Optional: For incubation with endosomal escape enhancers, add concurrently with the recombinant protein (0.1 mM chloroquine/80 mM sucrose).<br>5) Incubate cells for the desired duration (1 hour).<br>6) Add 3.6 μM Hoechst 33342 for 5 min to stain the nuclei.<br>7) Wash the cells thrice with DPBS containing 0.5 mg/mL heparin.<br>8) Wash the cells twice with DPBS.<br>9) Add 200 μL of fresh, pre-warmed medium per well.<br>10) Image immediately using ImageXpress® Pico automated cell imaging system.
**Critical Note:** Ensure that all washing notes are performed gently to avoid detaching the cell monolayer. Inadequate washing or excessive force can lead to cell loss and inconsistent signal quantification, particularly in high-content imaging assays.

Table S9. Protocol for the isolation and analysis of CPP-eGFP and SPΔCPP constructs for cellular uptake studies. Steps specific to this study are indicated in brackets. A critical note is denoted in bold.

Stage 1 – Identification of putative CPP motifs
1) Input the SP amino acid sequence of interest into CPPSite 2.0.<br>2) Run the prediction algorithm and evaluate high-scoring CPP candidates.<br>3) Cross-reference predicted motifs with existing literature to eliminate redundant or previously characterized sequences.
Stage 2 – Construction and isolation of CPP-eGFP fusion proteins
1) Design the constructs with the candidate motif fused to either the N- or C-terminus of eGFP.<br>2) Clone into the appropriate *E. coli* expression vector.<br>3) Express in *E. coli*, use the same procedure as in **Table S3** (no meat extract required).<br>4) Purify according **Table S4**, with the following modifications:<br>• Following Strep-column elution, exchange directly into DPBS, 10% Glycerol, pH = 7.2.<br>• Concentrate to ~2 mL using a spin concentrator with an appropriate MWCO.<br>• Perform gel filtration chromatography in DPBS, 10% Glycerol, pH = 7.2.<br>• Pool elution fractions and concentrate to final volume.<br>• Omit the LPS removal step.
**Critical Notes:** Ensure that buffer composition during gel filtration exactly matches the final storage buffer, as ionic strength and pH significantly affect CPP-eGFP stability.
Stage 3 – Live-cell imaging of CPP-eGFP constructs
1) Follow protocols outlined in **Table S6** (live-cell fluorescence microscopy) or **Table S7** (IFC).<br>2) Higher concentrations of CPP-GFP constructs of 8 – 15 μM are required (10 μM).
**Critical Note:** Cell survival at higher CPP-GFP concentrations should be tested (e.g., MTT assay) to rule out protein internalization due to compromised cell viability.
Stage 4 – Construction and isolation of SPΔCPP mutants
1) Design deletion constructs lacking the candidate CPP motif (SPΔCPP).<br>2) Clone into a bacterial expression plasmid.<br>3) Express in *E. coli* using the procedure in **Table S3**.<br>4) Purify using the standard workflow in **Table S4**.
Stage 5 – Live-cell imaging of SPΔCPP mutants

1) Follow the same protocols as in **Table S6** or **S7** respectively
2) Use standard concentrations applied to full-length CPP-FPs or SPs (4 µM).

Table S10. Co-immunoprecipitation protocol CPP-FP and SP with known binding partners. Steps specific to this study are indicated in brackets. Critical notes are denoted in bold.

Stage 1 – Cell seeding
1) Seed cells in dishes (NIH3T3, $3.5x10^6$ cells per 10 cm dish with a total volume of 15 mL culture media).
2) Use three dishes per condition.
**Critical Note:** Perform trial incubation with lower cell and protein amounts, check for cytoxicity.
Stage 2 – Sample incubation
1) Treat the cells with proteins in culture media for 1 hour (3 x 7 mL per condition, 1 µM each).
2) Wash the cells layer twice with 10 mL DPBS.
3) Treat the cells with 0.5 mg/mL heparin for 10 sec at RT.
4) Place plates on ice and wash twice with ice cold DPBS.
5) Scrape the cells with 1 mL ice-cold DPBS per dish, collect in a 1.5 mL tube.
Stage 3 – Cell lysis using a modified REAP method
1) Spin down at 16,000 × *g* for 10 sec at 4 °C, discard supernatant.
2) Dissolve the cell pellet in 900 µL ice-cold 0.1% NP-40 in PBS (1X PIC, 1X DTT).
3) Spin down at 16,000 × *g* for 10sec at 4°C, discard supernatant.
4) Re-suspend the pellet in 130 µL CoIP lysis buffer.
5) Sonicate the cells (Covaris sonicator) for 90 sec.
6) Incubate the nuclear isolates for 10 min, rotating at 300 rpm at 37 °C.
7) Add NaCl to a final concentration of 430 mM.
8) Sonicate the lysate (Covaris sonicator) for 20 sec.
9) Incubate the lysate rotating for 1 hour at 4 °C.
10) Centrifuge at 16,000 × *g* for 10 min at 4 °C, transfer supernatant to a new tube.
11) Add BME to a total concentration of 15 mM.
12) Dilute with CoIP buffer with 15 mM BME to a final NaCl concentration to 150 mM.
13) Centrifuge at 10,000 × *g*, for 20 min at 4 °C, transfer supernatant to a new tube.

14) Determine total protein concentration (Bio-Rad protein assay).

Critical Note: All steps should be performed in fast succession to ensure proper cellular sub-fractionation. Should pellets be observed following centrifugation steps, transfer the supernatants to a new tube before proceeding.

Stage 4 – Complex capture using Strep-Tag beads

1) For spiking experiments: Add recombinant protein as appropriate to a final concentration of 150 nM.
2) Take 20 µL per sample for western blotting (load).
3) Pre-wash the beads with 200 µL CoIP buffer with 15 mM BME per 1 µL of beads thrice, separating the beads from the solution by centrifugation at 500 rpm for 10 sec each time.
4) Add the sample(s) onto the beads (protein amount to be loaded should be no less 100 µg).
5) Vortex and incubate overnight with gentle rotation at 4 °C.
6) The next day, spin down the beads by centrifugation at 500 rpm for 10 sec each time.
7) Take 20 µL for western blotting (flowthrough).
8) Use 1 µL MagStrep“type 3”XT beads per 0.85 nmol of total protein to perform protein complex capture.
9) Wash the beads with 100 µL CoIP buffer with 15 mM BME per 1 µL of beads thrice, separating the beads from the solution solution by centrifugation at 500 rpm for 10 sec each time.
10) Add 6.7 µL of 4x sodium dodecyl sulphate (SDS) buffer supplemented with 10% (v/v) BME.
11) Heat the samples for 3 min at 95 °C.
12) Briefly spin down the samples.

Critical Note: Avoid disturbing the beads when aspirating liquid at each step. When centrifuging the beads, avoid speeds above 500 rpm.

Stage 5 – Western blotting

1) Load samples on SDS-PAGE gel (12% acrylamide), run as appropriate (80 V, 2 hours).
2) Blot as appropriate (iBlot® 2 Gel Transfer Device, program 0, 7 minutes).
3) Block the membranes (10 mL of Intercept® Blocking Buffer, 1 hour).
4) Add primary antibodies (anti-GFP (1:1000), anti-HDAC3 (1:1000), anti-β-tubulin (1:2000) in 5 mL Intercept® Antibody Diluent).
5) Incubate overnight with rotation at 4 °C.

6) Add secondary antibodies (1:20,000 in 5 mL Intercept® Antibody Diluent).

7) Incubate for 1 hour under gentle agitation at 4 °C.

8) Image the blots (Odyssey CLx imaging device).

9) Analyze (ImageStudio software, LI-COR Biosciences).

Critical Note: Check the nuclear fraction for cytosolic contamination by incubating it with a an antibody raised against β-tubulin, a known cytosolic marker.

References:

1. Beribisky, A.V., Steinkellner, H., Geislberger, S., Huber, A., Sarne, V., Christodoulou, J. and Laccone, F. (2022) Expression, Purification, Characterization and Cellular Uptake of MeCP2 Variants. *Protein J*, **41**, 345-359.
